# Intense Imagery Movements May Lead to Maladaptive Daydreaming: A Case Series and Literature Review

**DOI:** 10.1002/mdc3.14011

**Published:** 2024-03-27

**Authors:** Tammy Hedderly, Claire Eccles, Osman Malik, Farah Abdulsatar, Clare Mitchell, Tamsin Owen, Nirit Soffer‐Dudek, Claire Grose, Thomas V. Fernandez, Sally Robinson, Eli Somer

**Affiliations:** ^1^ Tic and Neurodevelopmental Movements Service (TANDeM), Children's Neurosciences, Evelina London Children's Hospital, Guys and St Thomas’ NHS Foundation Trust London UK; ^2^ Department of Women and Children's Health School of Life Course Sciences (SoLCS), King's College London London UK; ^3^ Leeds and York Partnership NHS Foundation Trust Leeds UK; ^4^ South London and Maudsley NHS Foundation Trust London UK; ^5^ Department of Paediatrics Schulich School of Medicine and Dentistry, University of Western Ontario London Ontario Canada; ^6^ Children's Health Research Institute, Lawson Health Research Institute, University of Western Ontario London Ontario Canada; ^7^ Department of Psychology Ben‐Gurion University of the Negev Beersheba Israel; ^8^ Child Study Center and Department of Psychiatry Yale University School of Medicine New Haven Connecticut USA; ^9^ Essex Child and Adolescent Mental Health Services, North East London NHS Foundation Trust Rainham UK; ^10^ Faculty of Social Welfare and Health Studies University of Haifa Haifa Israel

**Keywords:** intense imagery movements, maladaptive daydreaming, stereotyped behavior, default mode network, hyperphantasia

## Abstract

**Background:**

This case series highlights the connection between childhood intense imagery movements (IIM) and adult‐reported maladaptive daydreaming (MD). Motor stereotypies occur in typically developing children and also with co‐occurring neurodevelopmental differences. A subgroup with complex motor stereotypies reports accompanying intense imagery, often enhanced by the movements. This phenomenon can persist into adulthood and, in some cases, will need active management to prevent significant distress and impairment.

**Cases:**

Six adults, self‐reporting maladaptive daydreaming associated with stereotypies, are presented to demonstrate the associations.

**Literature Review:**

The clinical significance and function of IIM and MD are unclear, but several hypotheses are discussed, including the mechanism of emotional regulation through sensory seeking, as a process for processing childhood psychological trauma, as intrusive thoughts or images as part of a subtype of Obsessive Compulsive Disorder, or as a result of diverse attentional networks seen in neurodevelopmental disorders.

**Conclusions:**

This paper highlights important connections between IIM and MD. Many adults with MD show a childhood origin of stereotypical movements. Whilst immersive daydreaming may provide creativity and emotional regulation, there is evidence of distress and impairment of function for some adults, leading to MD diagnoses. Recognizing this phenomenon is important for all neurologists and physicians working with stereotypical movements.

Intense imagery movements (IIM) are observed in a distinct subgroup of children with complex motor stereotypies accompanied by intense imagery.^1^ Motor stereotypies are repetitive, rhythmical, and non‐purposeful movements ranging in complexity from simple actions such as rocking, tapping and flapping to more complex movements such as hand and arm twisting, pacing and jumping. They usually first present in infancy or early childhood and manifest during typical development in up to 60% of children, but also co‐occur in the context of neurodevelopmental diversity, eg, autism spectrum disorder (ASD), attention deficit/hyperactivity disorder (ADHD), obsessive‐compulsive disorder (OCD), and neurological disorders, eg, stroke, blindness.^2^ Children with IIM engage in deep thought accompanied by mental images whilst engaging in repetitive, driven, apparently non‐functional movements.^1^

In this case series, we highlight a connection between childhood IIM and the recently identified adult condition called maladaptive daydreaming (MD). MD involves engaging in compulsive, immersive daydreaming to the point of causing clinical distress, such as depression, shame, loneliness, and anxiety, and/or significant impairment in daily functioning, such as not completing tasks, not meeting work or social obligations, or losing relationships.^3^ As with IIM, MD can co‐occur with ASD, ADHD and OCD.^4^, ^5^

We are an international collaboration of clinicians and researchers in the field of movement disorders in children and dissociation in adults. For over a decade we have followed a cohort of several hundred children with IIM and, through clinical interviews and from assessments of correspondence with adult parents of children with IIM and adults with MD, we have observed that IIM is a precursor, for some children, to develop MD later in life. We use these excerpts, from direct interviews and from personal correspondence, with permission.

## Case Series

Case 1 (Male, 22): “I've been daydreaming since I was 4 or 5 years. I have to hold a pen/pencil, which I flip in my hands, or do a flip in the air and catch it. Without the pen/pencil in my hands, it doesn't work. It is combined with walking back and forth all the time. Music is also mandatory. Recently I had, what I call, an emotional breakdown, where I would drown in great guilt because I would realize/remind myself how much time and opportunity I waste every day. When I first started to daydream as a child, I did not use a pencil nor had anything in my hands. I was just walking back and forth (making faces/expressions), without the music. Later, I discovered that something in my hands boosts the experience of daydreaming. I told you that I play guitar and draw. When I used to daydream, I had very little time for both of my occupations because the daydreaming urge would hit again and again… when I finished with daydreaming, I would feel a peace of mind.”

Case 2 (Female, 19): “The earliest I can remember daydreaming was around five years where I would tell myself a story to go to sleep. Sometime around age 10–12, I got an MP3 player and began to daydream with music. At one point, I would basically be running up and down our hallway while daydreaming… The intensity of / need to daydream ultimately shifts with my mental health. The best I've gone was a week without getting an urge to daydream, when I first moved into my university dorms. When my mental health was at its worst, I would exit the classroom to go to my next class, immediately dissociate into a daydream, and then come out of it when I arrived at the next classroom. When daydreaming, I tend to move around, often tossing myself backwards as a result of some extreme action or motion being taken in the daydream. Often, I forget where I was physically situated, so on numerous occasions this leads me to banging my head onto walls and headboards.”

Case 3 (Male, 26): “I sometimes lost track of time and reality and found myself in a different reality in the middle of kindergarten or primary school classrooms and often woke up from reality through physical contact of someone breaking me from these sessions. I had truly vivid dreams back then, and often, I find myself suspecting whether some of my memories are creations of my own mind. For these cases, I had to reality check with people who were in these certain memories to make sure if it did happen or not. Some of these daydreaming sessions included smells, voices, and physical attributes (such as pain or sense of being touched). As I grew, the detail and the power of these daydreaming sessions decreased, I could no longer augment them into my reality, but they became more often. These rituals pretty much consisted of me repeatedly walking back and forth on a particular path in my room, or if it was summer, it would be on a large swing, swinging back and forth without any body effort and both cases under the influence of music according to the mood and setting of the daydreams. I have worlds, characters, stories, legends, epics, friends, lovers, and enemies that I often talk to… Everyone around me has been telling me I would be a great writer one day due to my imagination, but to be honest, I feel more like I am a chronicler who is just recording the events, scenes, and conversations happening before my eyes. I am currently 26 years old, moved in with my fiancé, working and studying for a master's abroad… Unlike what some would say, I never disliked my daydreaming condition. I have created so many unique people and places, that I do not want to let go of this ability as it honestly would feel like killing all of them. I don't want to lose it, not until I finish my stories.”

Case 4 (Female, 18): “I have been suffering from this for as long as I can remember, realistically, since the age of around four years old… I find myself daydreaming about 70% of my waking hours whilst socialising, but it rises to around 90% when alone. I spend a lot of time in my flat alone. When socialising, I often get frustrated when my daydreaming gets interrupted or just with the general fact that I will not be able to fantasise properly until I am alone again. I would prefer to daydream than be with friends and family, and this deeply upsets me. I would call it a compulsion. When I daydream, I zone out almost entirely from the outside world. I am still aware of my surroundings; I know what is real and what is not, but my daydreams are enticing and are my primary focus… Growing up in silence about this was difficult. I felt ashamed and weird and did not realize that there were others like me.”

Case 5 (Male, 46 years, Co‐occurring psychiatric or neurological diagnoses: None, Treatment: CBT, Neurofeedback): “My compulsion to daydream varies. Boredom and lack of purpose are the worst triggers for me. Immersive daydreaming can feed my ambitions and if I set clear goals in real life I am able to channel the problem‐solving and imaginative traits of daydreaming into academic and professional success. On the days when I am working towards a goal, I virtually do not daydream. Daydreaming is more intense but also much more pleasant while I am in motion. As a child I would stand up from the chair, pace along the hall and occasionally extend my mouth. Sometimes I want to jump out of my skin when I daydream and sit in a chair, but I have to supress it as it is not socially appropriate. So after some time I have to go for a walk. My best ideas come when I run 10k. The negative side of immersive daydreaming is the tendency to catastrophise as the imagination can extend into ruminating on the worst possible scenarios… My daughter is eight and has immersive daydreaming.”

Case 6 (Female, 46, No co‐occurring diagnoses, No treatment): “I've daydreamed intensely since a child. My two brothers have too and still do as adults. Movement is crucial, I used to click my jaw, rock and swing as a child, now I daydream when walking, on a train, twisting my hair, or pacing. I daydream visually, with strong emotions. They're repetitive but I change the endings. It has only ever been positive for imagining the future.”

## Literature Review

The function and clinical significance of IIM and MD require further research. One proposed hypothesis is that IIM/MD may function as a mechanism for regulating emotions, and some children report using IIM to self‐soothe.^4^ Some adults with MD report a childhood history of psychological trauma, and it is plausible that MD is a mechanism for the mind to adapt and respond to distressing experiences, however, many children exhibit stereotypies before the age of 2 years, and IIM/MD is not necessarily associated with adverse childhood experiences.^1^ MD may be a form of behavioral addiction or possibly a subtype of OCD.^6^, ^7^ Another possibility is that IIM/MD is linked to attentional problems such as those seen in ADHD.^8^

Although MD includes characteristics of behavioral addictions, obsessive‐compulsive spectrum symptoms, and attention problems, we have advocated for it to be considered a dissociative disorder,^9^ as it includes a rich inner world with semi‐agentic characters, a dual or “layered” state of consciousness, and a sense of detachment from reality.^9^ It is important to note that defining MD as a dissociative syndrome does not necessarily mean that trauma is implied. In recent years it is becoming evident that trauma explains only some, rather than all, of the variance of dissociation.^10^, ^11^, ^12^ For example, dissociation and decreased sense of agency both seem to play central roles in OCD, whereas trauma does not.^12^

There is currently a paucity of research exploring the neurobiological and cognitive mechanisms underlying IIM/MD. For most individuals, visual imagery plays a crucial role in daydreaming. The inability to form visual images (aphantasia) has been reported in 1% of the population, whilst the ability to create vivid images (hyperphantasia) has been reported in 3% of the population, with hyperphantasia associated with enriched autobiographical memory related to performance on measures of future‐directed imagination.^13^ Resting‐state functional magnetic resonance imaging (fMRI) revealed stronger functional connectivity between prefrontal regions and the visual–occipital network among hyperphantasic than aphantasic participants. The “visualization network” involves a combination of frontoparietal regions, attentional/executive control regions, and visual areas such as the fusiform and primary visual cortices.^13^ There is also an overlap with the default mode network (DMN). These findings suggest that the neural mechanisms underlying visual imagery and daydreaming involve complex interactions between various brain regions.

DMN refers to a functional system of brain structures that are active when individuals are in a resting state or performing easy or well‐practised tasks. Such brain activity involves the medial prefrontal cortex, posterior cingulate cortex, inferior parietal lobe, middle temporal lobe, and precuneus.^14^ It is implicated in cognition domains critical for self‐generated thought and images.^15^ In the context of IIM/MD, some individuals seem to be highly engaged in scripting elaborate inner scenarios, while others appear “zoned out” or off‐task. One hypothesis is that the DMN is active during stereotyped movement and that stereotypies may be associated with daydreaming through the activation of the DMN's role in retrieving episodic, autobiographical, or semantic knowledge, reappraising emotional information, self‐reflection, and future thinking. Future research using comparative brain imaging techniques is planned with an aim to determine whether the mental tasks of scripting and developing MD plots are “off‐task” or “on‐task” activities.

## Discussion

This case series highlights novel clinical observations about adults with MD. Firstly, all six cases highlight the childhood origin of IIM/MD. Secondly, five of the representative cases describe an association in adults between stereotyped movements and daydreaming. For example, Case 1 describes hand movements flipping a pen/pencil and pacing; Case 2 describes moving around according to the action in the daydream and head banging; Case 3 describes pacing and swinging; Case 5 describes walking and running; Case 6 describes walking, hair twisting and pacing. Many adults who continue to have stereotypies have learnt to privatize them by supressing or only engaging in movements when alone. The repetitive movements of motor stereotypies appear to play an essential role in MD by amplifying the immersive nature of daydreaming, by enhancing focus through self‐hypnotic qualities (perhaps similar to the swinging of a pendulum which may help induce a hypnotic state of consciousness), and through embodying the actions imagined by characters within the daydream. More research is needed to better understand the mind–body association responsible for the absorptive effect created by the stereotypical movement. Possibly, repetitive movement may bring about a habituation and decrease in external sensory input, enabling the daydreamer to engage more fully with internal events. Music also appears to play an important role alongside movement for some, as Cases 1, 2, and 3 highlight.

Thirdly, the imagery that persists into adulthood can have more adult‐oriented themes, and often appears to enhance creativity, productivity or artistic talent. Case 1 plays the guitar and is an artist; Case 3 speaks about the association between daydreaming and writing with enhanced imagination; Case 5 has his “best ideas” whilst running and daydreaming; and Case 6 “solves problems”. For some adults with intense visual imagery connected to stereotypies, the experience can be pleasurable and provide benefits. However, in adults with MD, there is distress and a significant adverse impact on wellbeing.

Our final observation of this case series is the contrast between the often‐enjoyable experience of IIM in childhood and the impact of MD on function and mental health in adults. Case 1 describes satisfaction from daydreams but also guilt due to the time and opportunity cost of daydreaming; Case 2 highlights how MD intrudes into their reality, with self‐injury and banging their head into objects; Case 4 speaks about the length of time spent alone, the proportion of time lost to daydreaming, the compulsive nature, plus the extreme shame felt; Case 5 describes catastrophising and rumination and has sought psychological therapy. Dysfunction is not however inevitable as illustrated in Cases 3 and 6 who have never disliked the daydreaming condition. It is likely that most young children who have this ability enjoy it, while it may become impairing over time as demands from the outside world grow.

In conclusion, we present preliminary clinical observations connecting childhood IIM to adult‐reported MD in a subgroup of individuals. Further observational research is planned to better understand the natural history of IIM/MD, to help define the diagnostic criteria for MD, and to develop probable indicators for those at risk of MD. We are working together to further understand pathophysiological mechanisms, including the likely complex neurogenetics, and the role of developmental stage and life events in the progression to MD. We hope to raise awareness for movement disorder clinicians, neuropsychiatrists or psychologists, and neurocognitive researchers, as well as for the wider community of those experiencing IIM/MD so that the appropriate support and management can be accessed when necessary.

## Author Roles

(1) Research project: A. Conception, B. Organization, C. Execution; (2) Statistical Analysis: Not applicable; (3) Manuscript Preparation: A. Writing of the first draft, B. Review and Critique.

TH: 1A, 1B, 1C, 3A, 3B

CE: 3A, 3B

ES: 1A, 1B, 1C, 3B

FA: 3B

CM: 1C, 3B

TO: 1C, 3B

NS‐D: 3B

CG: 1C, 3B

TF: 3B

SR: 1C, 3B

OM: 1A, 1B, 1C, 3A, 3B.

## Disclosures

**Ethical Compliance Statement:** Patient consent for inclusion in publication was given in writing by patients to authors ES (Cases 1–4) and TH (Cases 5 & 6). We confirm that we have read the Journal's position on issues involved in ethical publication and affirm that this work is consistent with those guidelines.

**Funding Sources and Conflicts of Interest:** No specific funding was received for this work. The authors declare that there are no conflicts of interest relevant to this work.

**Financial Disclosures for Previous 12 months:** NS‐D holds an Israel Science Foundation grant on the dissociative mechanisms of Maladaptive Daydreaming. TF holds two NIH/NIMH grants “Neurofeedback from the supplementary motor area for Tourette's Syndrome” and “2/7‐Collaborative Genomic Studies of Tourette's Disorder”; and a grant from Foundation for OCD Research / New Venture Fund “Breaking through OCD Genetics”; DLA Piper (Law Firm): Paid consultant for expert testimony related to potential causes of autism spectrum disorder and ADHD. FA holds a CHRI Internal Research Grant Fund.

## References

[1] Robinson S , Woods M , Cardona F , Baglioni V , Hedderly T . Intense imagery movements: a common and distinct paediatric subgroup of motor stereotypies. Dev Med Child Neurol 2014;56(12):1212–1218. 24947872 10.1111/dmcn.12518

[2] Evans DW , Gray FL , Leckman JF . The rituals, fears and phobias of young children: insights from development, psychopathology and neurobiology. Child Psychiatry Hum Dev 1999;29:261–276. 10422351 10.1023/a:1021392931450

[3] Somer E . Maladaptive daydreaming: a qualitative inquiry. J Contemp Psychother 2002;32:197–212.

[4] Somer E , Soffer‐Dudek N , Ross CA , Halpern N . Maladaptive daydreaming: proposed diagnostic criteria and their assessment with a structured clinical interview. Psychol Conscious Theory Res Pract 2017;4(2):176–189.

[5] Theodor‐Katz N , Somer E , Hesseg RM , Soffer‐Dudek N . Could immersive daydreaming underlie a deficit in attention? The prevalence and characteristics of maladaptive daydreaming in individuals with attention‐deficit/hyperactivity disorder. J Clin Psychol 2022;78(11):2309–2328. 35355262 10.1002/jclp.23355 PMC9790222

[6] Pietkiewicz IJ , Nęcki S , Bańbura A , Tomalski R . Maladaptive daydreaming as a new form of behavioral addiction. J Behav Addict 2018;7(3):838–843. 30238787 10.1556/2006.7.2018.95 PMC6426361

[7] Salomon‐Small G , Somer E , Harel‐Schwarzmann M , Soffer‐Dudek N . Maladaptive daydreaming and obsessive‐compulsive symptoms: a confirmatory and exploratory investigation of shared mechanisms. J Psychiatr Res 2021;136:343–350. 33636690 10.1016/j.jpsychires.2021.02.017

[8] West MJ , Somer E , Eigsti IM . Immersive and maladaptive daydreaming and divergent thinking in autism Spectrum disorders. Imagin Cogn Pers 2023;42(4):372–398. 38031581 10.1177/02762366221129819 PMC10686311

[9] Soffer‐Dudek N , Somer E . Maladaptive daydreaming is a dissociative disorder: supporting evidence and theory. In: Dorahy MJ , Gold SN , O'Neil JA , eds. Dissociation and the Dissociative Disorders: Past, Present, Future. Vol 87. 2nd ed. Routeledge, New York; 2022:295–309. 10.1111/jopy.12391.

[10] Lynn SJ , Polizzi C , Merckelbach H , Chiu C‐D , Maxwell R , van Heugten D , Lilienfeld SO . Dissociation and dissociative disorders reconsidered: beyond sociocognitive and trauma models toward a transtheoretical framework. Ann Rev Clin Psychol 2022;18:259–289. 10.1016/j.psychres.2013.04.017. 35226824

[11] Michal M , Adler J , Wiltink J , et al. A case series of 223 patients with depersonalization‐derealization syndrome. BMC Psychiatry 2016;16:203. 10.1016/j.janxdis.2017.02.005. 27349226 PMC4924239

[12] Soffer‐Dudek N . Obsessive‐compulsive symptoms and dissociative experiences: suggested underlying mechanisms and implications for science and practice. Front Psychol 2023;14:1132800. 10.3389/fpsyg.2023.1132800. 37051604 PMC10084853

[13] Milton F , Fulford J , Dance C , et al. Behavioral and neural signatures of visual imagery vividness extremes: Aphantasia versus Hyperphantasia. Cereb Cortex Commun 2021;2(2):tgab035. 34296179 10.1093/texcom/tgab035 PMC8186241

[14] Broyd SJ , Demanuele C , Debener S , Helps SK , James CJ , Sonuga‐Barke EJS . Default‐mode brain dysfunction in mental disorders: a systematic review. Neurosci Biobehav Rev 2009;33(3):279–296. 18824195 10.1016/j.neubiorev.2008.09.002

[15] Andrews‐Hanna JR , Smallwood J , Spreng RN . The default network and self‐generated thought: component processes, dynamic control, and clinical relevance. Ann N Y Acad Sci 2014;1316(1):29–52. 24502540 10.1111/nyas.12360 PMC4039623

